# Prediction of individual graft for anterior cruciate ligament reconstruction using anthropometric data

**DOI:** 10.1007/s00402-022-04682-0

**Published:** 2022-11-04

**Authors:** Patrick Sadoghi, Veronika Röggla, Hannes Beiglböck, Benjamin Schett, Martin Reschl, Stefan Fischerauer, Patrick Reinbacher, Harald K. Widhalm

**Affiliations:** 1 Department of Orthopaedics and Trauma, Medical University of Graz, Auenbruggerplatz 5, 8036 Graz, Austria; 2 Department of Trauma Surgery, Medical University of Vienna, Währinger Gürtel 18-20, 1090 Vienna, Austria

**Keywords:** ACL, MRI, Anthropometry, Individual, Reconstruction

## Abstract

**Introduction:**

Multiple options for individual anterior cruciate ligament (ACL) reconstruction exist; still, there are no guidelines for the preoperative preparation. The aim of this study was to assess the correlation between patients’ anthropometric data (height, weight, and age) and measurements of potential tendons (quadriceps-, patella, hamstrings tendon) for an anterior cruciate ligament reconstruction.

**Material and methods:**

MR images of 102 patients have been analyzed. Measurements of the ACL were performed with respect to its length and angle. The diameter and length as well as width of the quadriceps and patella tendon, the cross-sectional area (CSA) and diameter of the hamstring tendons have been assessed. Patients’ height, weight, BMI, sex and age have been recorded. The correlations of these measurements with the patients’ anthropometric data have been calculated. Inter-rater and intra-rater reliability based on intra-class correlation (ICC) was evaluated.

**Results:**

The mean lengths of the ACL were 29.8 ± 3.5 mm, tibial insertion sites 15.8 ± 2.5 mm and femoral insertion sites 15.2 ± 3.0 mm. Thickness of the quadriceps tendons was 4.7 ± 1.1 mm and patella tendon 3.2 ± 0.7 mm. The patients’ height showed significant positive correlations with the CSA of the hamstring tendon measurements, the length of the ACL, and the insertion sites of the ACL. Patients’ weight showed significant positive correlations with patella tendon thickness, the CSA of the hamstring tendons, the length of the ACL, and the tibial and femoral insertion sites. Patients’ age showed a significant positive correlation with patella tendon thickness. The ICCs for intra- and inter-rater reliability were 0.98 (95% CI 0.95–0.99, *p* < 0.001) and 0.94 (95% CI 0.88–0.99, *p* < 0.001).

**Conclusion:**

Anthropometric data with respect to height, weight, and sex can help to predict the dimension of tendons for ACL reconstruction and do correlate with ACL tendon. Patients at risk for small graft dimensions and failure are younger than 20 years and physically active. MRIs of patients at risk for small graft dimensions should be analyzed on tendon length and cross section areas preoperatively to determine the appropriate tendon harvest and fixation technique.

## Introduction

The rupture of the anterior cruciate ligament (ACL) is one of the most common injuries of the knee joint [24]. ACL reconstruction techniques developed tremendously in the last decades, so nowadays the reconstruction is mostly preferred [30]. Detailed preoperative planning, including MRI measurements, leads to the best outcome for the patient [7, 15, 35]. The hamstring tendons as well as the patella tendon are the most discussed autografts in the literature [12, 25, 31]. While the quadriceps tendon is mostly used for revision surgeries, it gains more and more popularity even for primary reconstructions [11].

If the quality of the graft is not sufficient, there is evidence for an increased rate of re-ruptures with consequences such as revision surgery, prolonged physiotherapy, and more time to reach the same sport level as before.

The individual anatomic anterior cruciate ligament reconstruction is described as a decision for one technique, that considers the history of the patients’ knee, its anatomical and functional situation, results of clinical examination, and the patients’ demands with respect to the future sport career [4, 23]. The goal is to reproduce the native anatomy in the knee joint [29]. Therefore, all tendons, which can be used as autografts, should be considered. An MRI helps both, to evaluate the situation in the knee joint and to measure the size of possible autografts [21]. By being aware of the patients’ anatomy, the surgeon can individually evaluate the tendon harvest and opt for the preferred technique. Many papers investigated the advantages and disadvantages of each graft option [1, 2, 17, 18, 20, 38].

It has also been confirmed that MRI and intraoperative measurements of tendons achieve good comparability [6, 15, 36].

In 2013, Thomas et al. evaluated the influence of anthropometric data on hamstring grafts [32]. The patients’ height (*r* = 0.38, *p* < 0.01) and weight (*r* = 0.29, *p* < 0.01) showed positive correlations with the size of the harvested hamstring tendons. However, in this study, no MR images were involved and exclusively the hamstring tendons have been investigated with lack of evaluation of the patella tendon and the quadriceps tendon.

Thus, the aim of the study was to investigate the relationship of anthropometric data and relevant tendons (quadriceps, patella, hamstrings) that can be used as autografts to improve the preoperative planning in order to achieve best clinical outcome after performing anatomical individualized ACL reconstruction.

The hypothesis of this study was that anthropometric data such as sex, height, weight, and age correlate with metric properties of anterior cruciate, quadriceps, patella, and hamstring tendons.

## Patients and methods

**Fig. 1:**
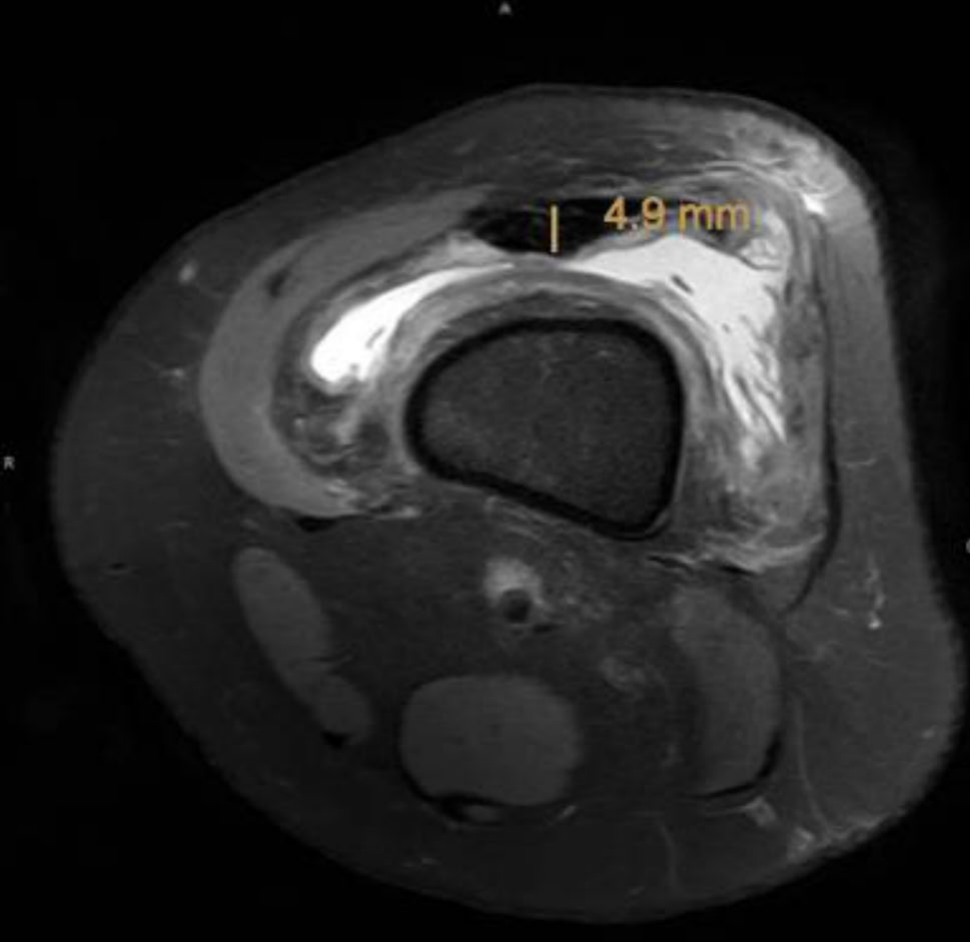
Thickness of the quadriceps tendon (QT) on an axial MRI (T2 sequence) measured 2 cm above the insertion of the tendon at the cranial patella pole

**Fig. 2:**
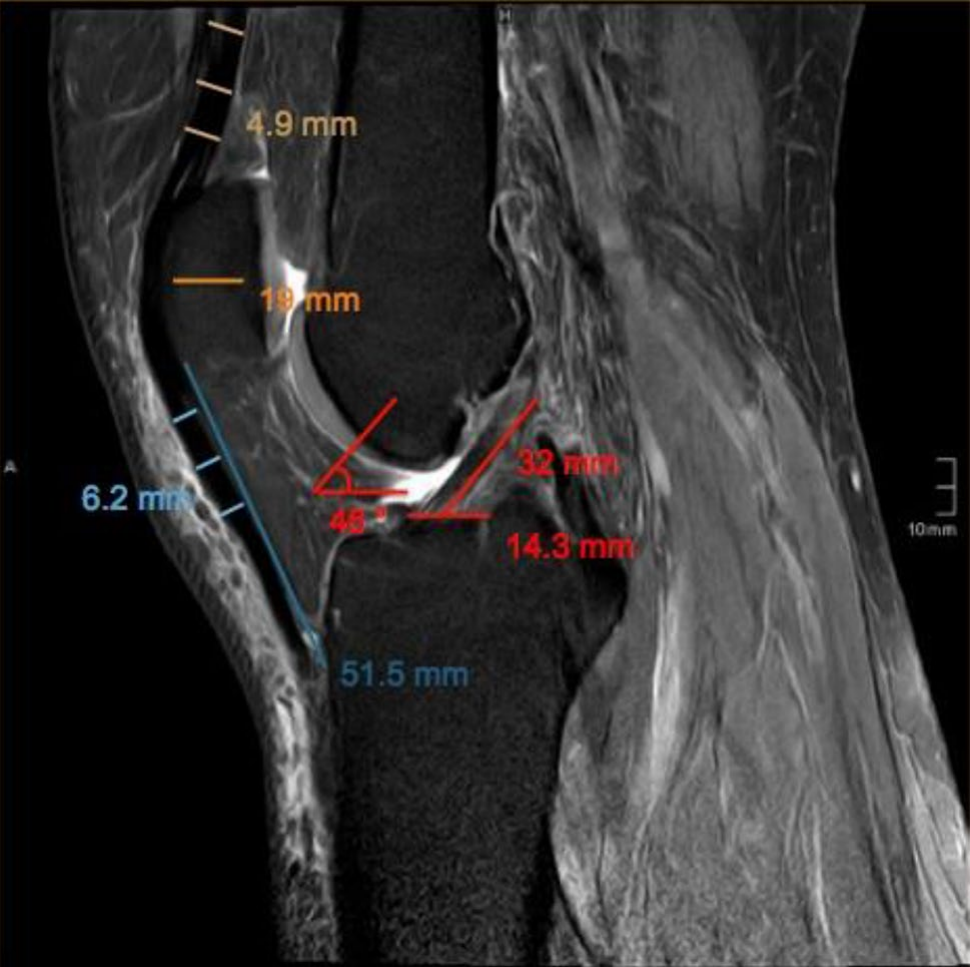
Measurements of the thickness of the quadriceps tendon (QT), patella, length of the patella tendon (PT), thickness of the patella tendon, length and angle of the anterior cruciate ligament (ACL) and tibial insertion site of the ACL on a sagittal MRI (T2 sequence)

The presented study followed accepted ethical, scientific and medical standards and was conducted in compliance with recognized international standards, including the principles of the Declaration of Helsinki. It was approved by the institutional review board (1306/2015) and has been performed as a retrospective single-center data analysis using collected data. MR images of 102 patients´ knees, performed between 2008 and 2015, were included in the study. The mean patients’ age was 39.9 ± 18.9 (range 11.0–79) years, and 50 patients were female, and 52 male. All patients, who already had undergone surgery at the certain knee, patients with anterior cruciate ligament ruptures or severe injuries and patients with incomplete MR images have been excluded. The MRI was performed on a special 3-Tesla MRI device (Achieva MRI-device in the general hospital, E08MRD) at the study center using T2 sequences within 3 months after the accident. The MRIs have been screened retrospectively for possibly presence of pathologies according to cartilage, meniscus, ACL, and PCL by a board-certified orthopedic and trauma surgeon. The patients’ weight, height, age, and sex were recorded from the written consent form of the MRI or the medical record, and the body mass index (BMI) was calculated. Detailed measurements of the ACL (length, length of the insertion sites, and angle of the ACL) have been performed. Moreover, diameter, length as well as width of the quadriceps and patella tendon, as well as the cross-sectional area (CSA) and diameter of the hamstring tendons were calculated, respectively. Figures1, 2 the quadriceps and patella tendon was measured in 10, 20, and 30 mm distance from the patella. For the diameter of the hamstring tendons, the axial sequence, which showed both the most circular and largest semitendinosus tendon (ST), was used. The same image was used for the gracilis tendon (GT). The freehand region-of-interest (ROI) tool was used to measure the cross-sectional area (CSA) of the ST and GT. Two observers independently measured the certain structures, using the T2-sequence of the MRIs. They were blinded to any information about the patient and the assessment of the other observer.

### Statistical analysis

Descriptive statistics, including mean, minimum, maximum, quartiles, and standard deviation, were calculated for all anthropometric and radiological variables. The Spearman correlation coefficient was used to assess the correlation between anthropometric data and tendon measurements. We calculated inter- and intra-rater reliability based on intra-class correlation for the radiological measurements. The *t* test analysis was used to test mean differences of the tendons between females and males. Unadjusted *p* values were reported for the correlation coefficient and the *t* tests. Subsequently, the Bonferroni–Holm correction was used to adjust for multiple testing. In a post hoc power analysis, based on the difference of the CSA of the semitendinosus tendon between females (*n* = 50) and males (*n* = 52), with an alpha-level of 5%, a power greater than 80% was achieved.

## Results

Descriptive statistics of 102 patients (50 females and 52 males) who were included in our study are illustrated in Table 1. The mean values of relevant structures (quadriceps- and patella tendon, CSA of ST and GT and ACL measurements) are given with minimum and maximum values and illustrated in Table 2.

**Table 1:** Descriptive statistics of anthropometric data and the patients’ age in female and male patients

	Male (SD)	Female (SD)	Mean (SD)	*p* value
Age (years)	35.6	34.2	34.9 (18.9)	NS
Height (cm)	177.2	165.4	171.4 (0.1)	NS
Weight (kg)	78.5	63.9	71.4 (17)	NS
BMI (kg/m2)	24.5	23.4	24 (4.4)	NS

**Table 2:** Descriptive statistics of the measurements of the tendons and the anterior cruciate ligament

	Mean (mm)	SD (mm)	Minimum (mm)	Maximum (mm)
QT-Thickness	4.8	1.1	2.0	9.2
PT-Length	48.3	6.8	30.0	65.4
PT-Thickness	3.2	0.7	1.7	5.7
ST-Diameter	4.8	0.8	2.9	7.1
ST-CSA	13.4	3.6	6.2	27.0
GT-Diameter	4.0	0.8	2.7	8.2
GT-CSA	7.8	2.0	4.0	14.1
ACL-Length	29.8	3.5	22.9	41.4
Tibial Insertionsite	15.8	2.5	10.4	27.8
Femoral Insertionsite	15.2	3.1	9.3	23.0

Several positive correlations have been observed. The strongest correlation has been detected between the length of the ACL and the patients’ height (*r* = 0.619, *p* < 0.001).

Between male and female patients, there was a significant difference of the CSA of the ST (mean difference = 3.11 mm^2^, 95% CI 1.8–4.3 mm^2^, *p* < 0.001) and the GT (mean difference = 1.37 mm^2^, 95% CI 0.6–2.1 mm^2^, *p* < 0.001), the length of the ACL (mean difference = 3.45, 95% CI 2.24–4.66 mm, *p* < 0.001), and the tibial (mean difference = 1.92, 95% CI 1.0–2.8 mm, *p* < 0.001) and femoral (mean difference = 2.41, 95% CI 1.3–3.5 mm, *p* < 0.001) insertion site. The patients’ age and the thickness of the patella tendon (*r* = 0.298, *p* = 0.002) showed a significant correlation. After the Bonferroni–Holm correction, the BMI did not correlate with any measurements. All correlations are illustrated in Table 3.

**Table 3:** Correlation analysis of anthropometric data (height, weight, BMI, sex, age) with autografts for anterior cruciate ligament reconstruction (quadriceps, patella, semitendinosus, and gracilis tendons)

	Height	Weight	BMI	Sex	Age
QT-thickness	NS	NS	NS	NS	NS
PT-thickness	NS	*r* = 0.37 (*p* < 0.001)	NS	NS	NS
ST–CSA	*r* = 0.37 (*p* < 0.001)	*r* = 0.43 (*p* < 0.001)	NS	*r* = − 0.44 (*p* < 0.001)	NS
GT–CSA	*r* = 0.35 (*p* < 0.001)	NS	NS	*r* = − 0.34 (*p* < 0.001)	NS
ACL length	*r* = 0.62 (*p* < 0.001)	*r* = 0.51 (*p* < 0.001)	NS	*r* = − 0.49 (*p* < 0.001)	NS
Tibia insertion site	*r* = 0.42 (*p* < 0.001)	*r* = 0.36 (*p* < 0.001)	NS	*r* = − 0.38 (*p* < 0.001)	NS
Femoral insertion site	*r* = 0.54 (*p* < 0.001)	*r* = 0.49 (*p* < 0.001)	NS	*r* = − 0.40 (p < 0.001)	NS

**Fig. 3:**
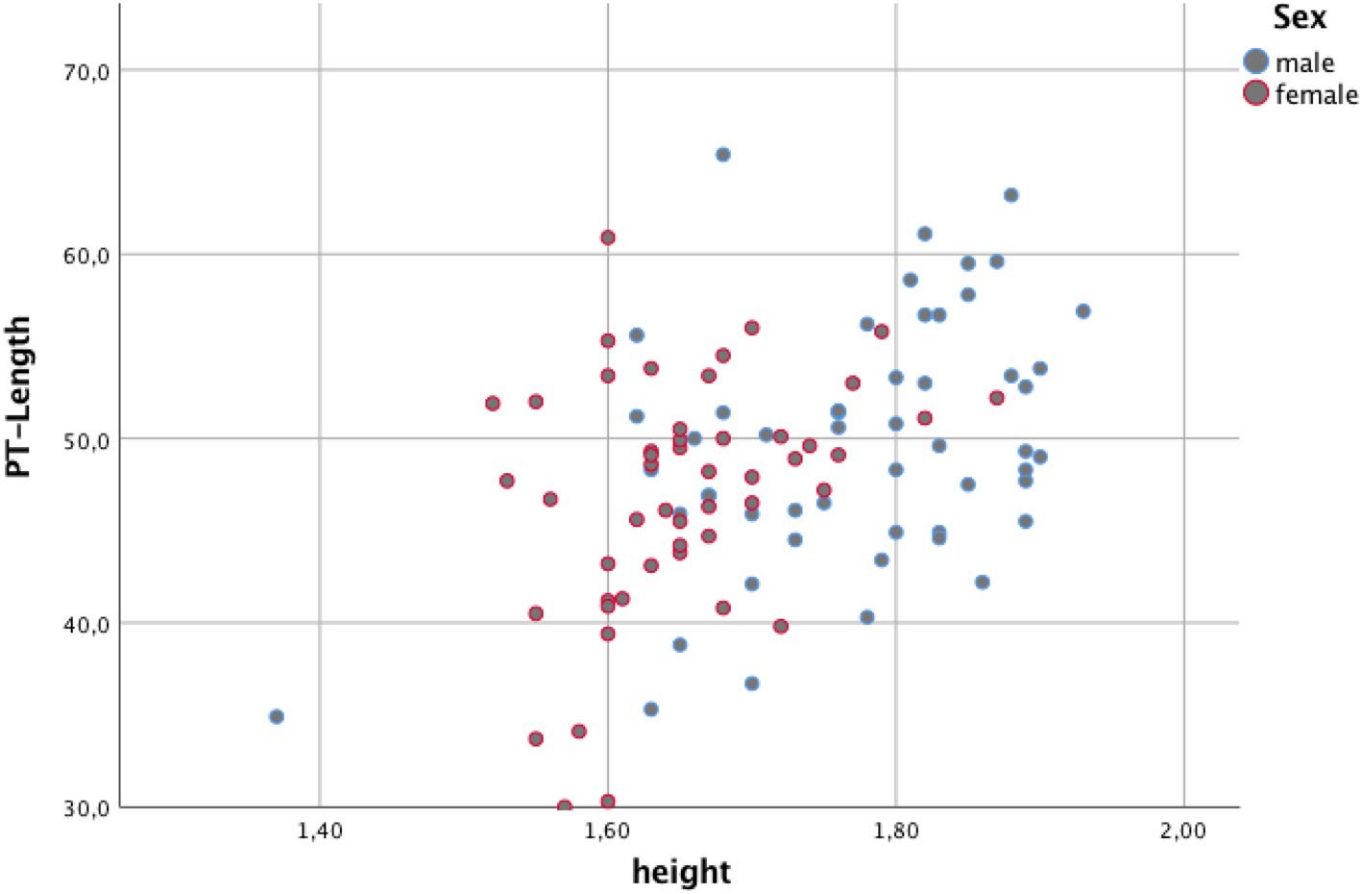
Correlation analysis between patients’ height and length of patella tendon (PT) for female and male patients

**Fig. 4:**
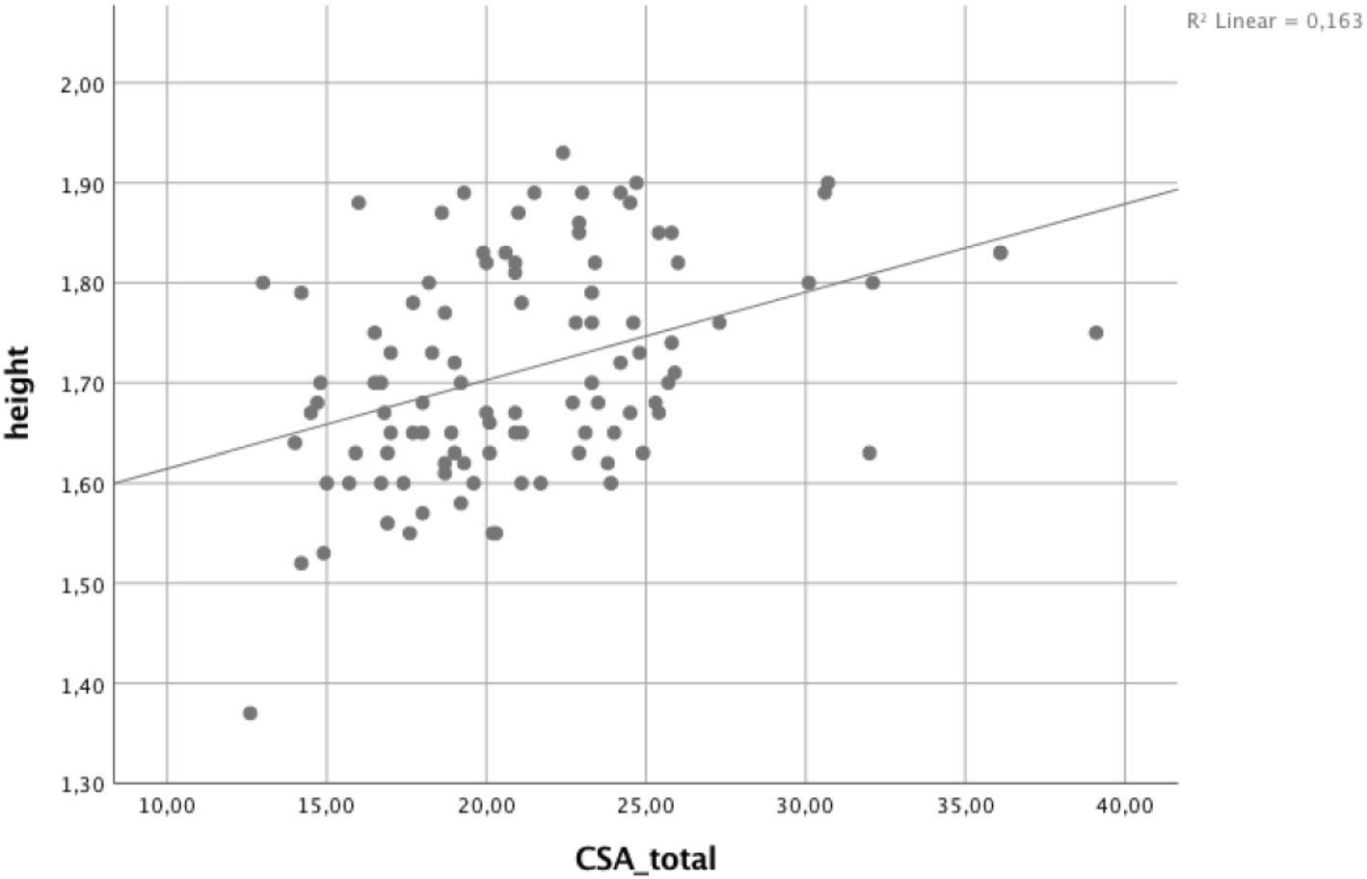
Correlation analysis between patients’ height and cross-sectional area (CSA)

**Fig. 5:**
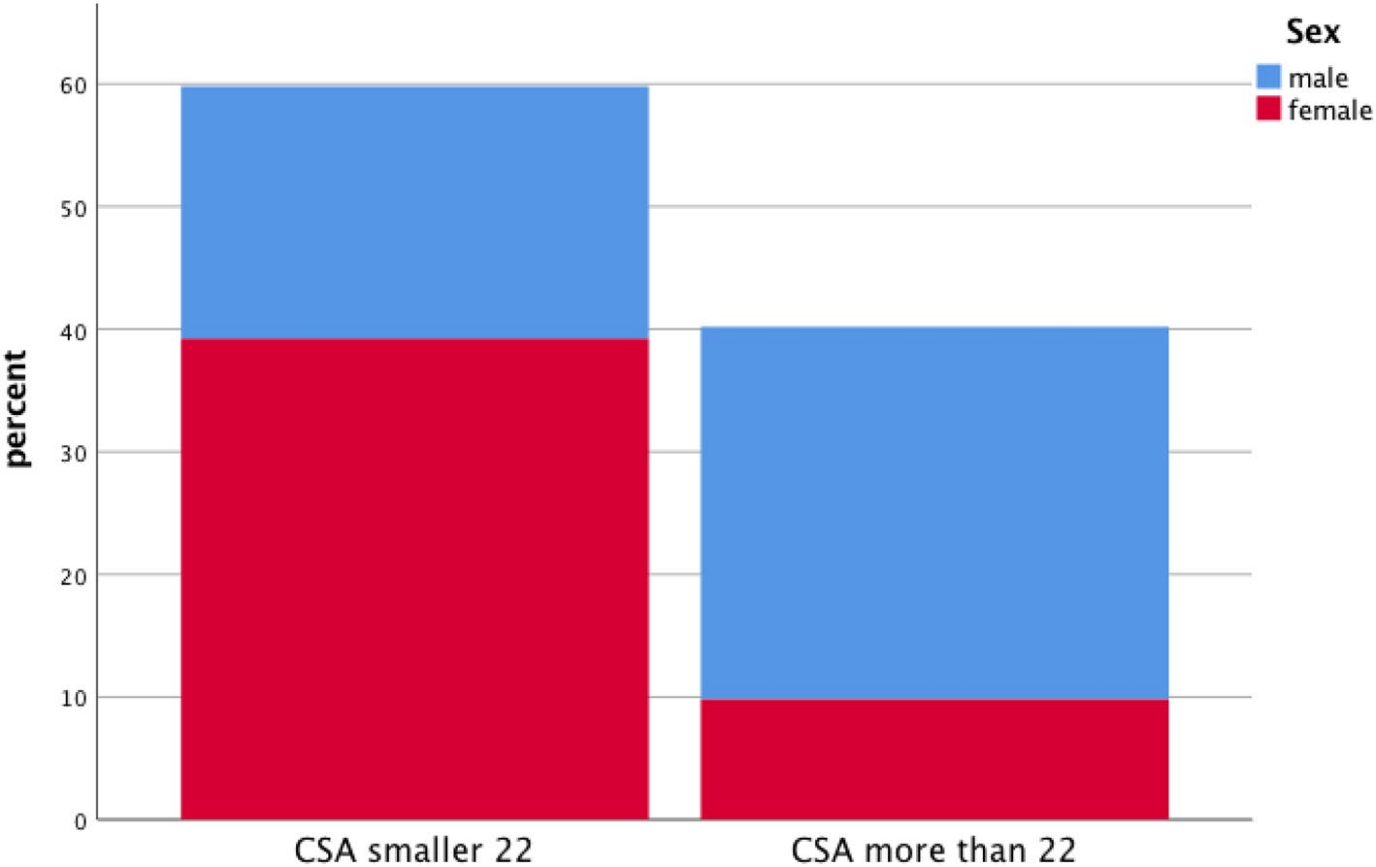
Cross-sectional area (CSA) limit of 22 mm^2^, which predict a good autograft, depending on sex

Moreover, results are given in Figs. 3, 4, showing sex-dependent correlations between PT-length as well as CSA total and patient’s height Figs. 3, 4. In addition, Fig. 5 is showing percentage of measured CSA lower and higher than 22 mm^2^ and the impact on sex.

**Fig. 6:**
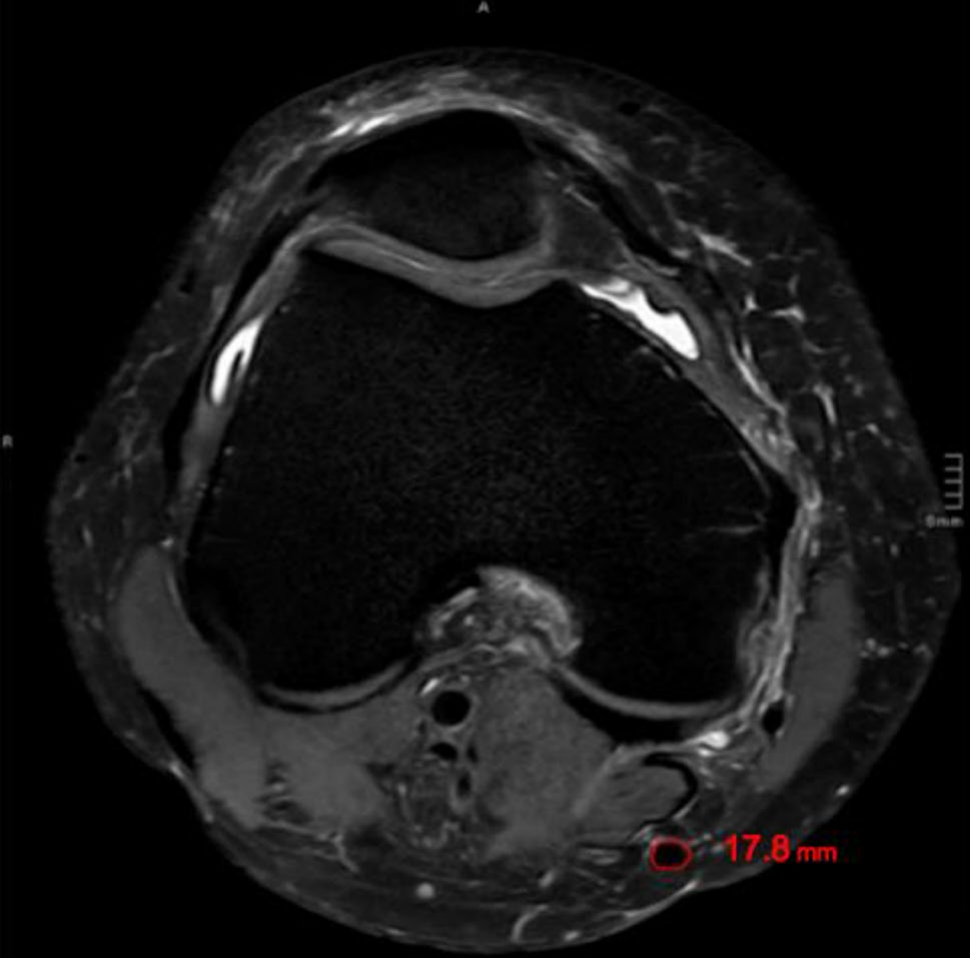
Cross-sectional area (CSA) of the semitendinosus tendon (ST) on an axial MRI (T2 sequence) of a patient of 115 kg and a calculated CSA of the semitendinosus tendon of 17.8 mm^2^

**Fig. 7:**
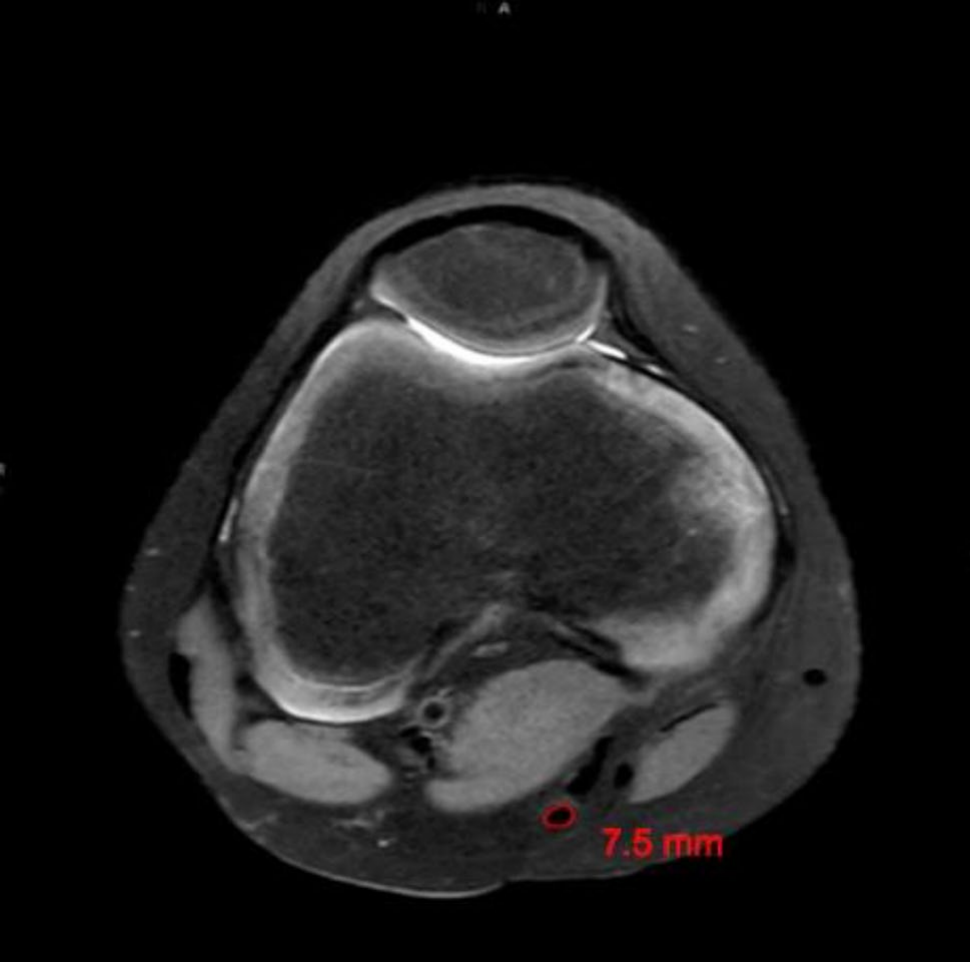
Cross-sectional area (CSA) of the semitendinosus tendon (ST) on an axial MRI (T2 sequence) of a patient with a weight of 35 kg showing a CSA of the semitendinosus tendon of 7.5 mm^2^

Figures 6, 7 show an exemplary comparison of the CSA-sizes of the ST. In Fig. 1, the patients’ weight is 115 kg and the CSA of the semitendinosus tendon is 17.8 mm^2^ versus; in Fig. 2, the patients’ weight is 35 kg and the CSA of the ST is 7.5 mm^2^.

The inter-rater reliability between the radiological measurements based on intra-class correlation (ICC) was strong (ICC = 0.94, 95% CI 0.88–0.99, *p* < 0.001). The intra-rater reliability also proved strong conformity (ICC = 0.98, 95% CI 0.95–0.99, *p* < 0.001). Detailed numbers concerning the inter- and intra-rater reliability are listed in Table 4.

**Table 4:** Inter- and intra-rater reliability for measurements of the knee in the MRI (quadriceps, patella, semitendinosus, gracilis tendon and ACL)

	Inter-rater reliability (95% CI)	Intra-rater reliability (95% CI)
QT-Thickness	0.946 (0.92–0.964)	0.999 (0.998–1.000)
PT-Thickness	0.924 (0.888–0.949)	0.997 (0.993–0.999)
ST-CSA	0.993 (0.990–0.995)	0.990 (0.978–0.996)
GT-CSA	0.979 (0.97–0.986)	0.978 (0.95–0.99)
ACL-length	0.964 (0.947–0.976)	0.979 (0.951–0.991)
tibial insertion site	0.877 (0.818–0.917)	0.945 (0.875–0.976)
femoral insertion site	0.913 (0.872–0.941)	0.964 (0.918–0.984)

## Discussion

The aim of the study was to investigate the relationship of anthropometric data and relevant tendons (quadriceps, patella, hamstrings) that can be used as autografts to improve the preoperative planning for anatomical individualized anterior cruciate ligament reconstruction. The hypothesis of this study was therefore that anthropometric data such as sex, height, weight, and age influence the lengths and cross section areas of tendons, which can be used for individual ACL surgery. We found in our study that anthropometric data with respect to height, weight, and sex have a moderate correlation with the size of tendons for ACL reconstruction (quadriceps, patella, semitendinosus, and gracilis tendon). The MRI-based measurements revealed good to excellent inter- and intra-class correlations.

To our knowledge, there exists few evidence concerning a potential correlation of MRI, anthropometric data, and the length and thickness of tendons, which can be used for ACL reconstruction. However, there are several studies, which measured certain tendons intra-operatively and then correlated their findings with anthropometric data [5, 9, 26, 32, 33, 37]. The advantage of MRI data that include all 4 tendons is that the preferred graft choice can be preoperatively evaluated for a dimensional fit in respective to patient’s anthropometrics.

Anthropometric data have been identified to have an impact on specific anatomical structures in previous investigations [3, 8, 21, 27, 32, 34, 39]. Some authors describe that body height reveals the greatest influence on the size of tendons [5, 13, 16, 26, 34] and other authors state body weight as the strongest indicator [21, 33].

Tuman et al. tried to find an accurate factor to predict the diameter of hamstring tendons for the ACL reconstruction [34]. The most important result was the moderate correlation between the patients’ body height and the diameter of hamstring tendons (*r* = 0.36, *p* < 0.001). Zakko et al. described the correlation of quadriceps and patella graft diameter with body height, weight, BMI and sex [39]. Weak to moderate correlations were detected between quadriceps tendon thickness and height (*r* = 0.3), weight (*r* = 0.3), BMI (*r* = 0.3), and sex (*r* =  − 0.4) and patellar tendon thickness and height (*r* = 0.4), weight (*r* = 0.3), and sex (*r* =  − 0.4). There were no correlations between the age and measurements of the tendons.

Thomas et al. [32] investigated the correlation between anthropometric data and tendons that can be used for ACL reconstruction in 121 patients. The study showed that the patients’ body weight (*r* = 0.29, *p* < 0.01) and height (*r* = 0.38, *p* < 0.01) influence the size of the graft diameter and that the BMI was not statistically significant. Sex and graft diameter showed a significant correlation (*r* = − 0.28, *p* < 0.01) with the graft being thicker in male patients [27]. Thomas et al. further found that patients with a height less than 125 cm have a high risk of harvesting a graft with a diameter less than 7 mm and propose that an alternate graft-technique should be used [32]. Another study stated that if a patient is less than 147 cm tall, it is likely to harvest a quadruple hamstring graft with a diameter less than 7 mm [34]. To avoid a re-rupture the recommended diameter of the chosen graft should be no less than 7 mm [13]. According to the paper of Grawe et al. showing that CSAs of combined semitendinosus and gracilis tendons > 22 mm^2^ provide a fourfold graft diameter of > 8 mm^2^. [14] In our study, it could be shown that combined CSA values higher than 22 mm^2^ were seen in approximately 40% of the cases. As CSA values showed a high correlation to body height, the median CSA threshold > 22 mm^2^ was observed at heights larger than 175 cm. The association of body height and sex revealed an underrepresented prevalence of combined CSA values > 22 mm^2^ in women of our study group. Grafts larger than 8 mm were described to provide a protective effect in patients aged younger than 20 years, a group identified at risk for failure. In these patients, anthropometric parameters and imaging (MRI and ultrasound) can be used preoperatively to predict hamstring autograft diameter and surgeons might opt for patella tendon autografts instead [16].

The variable of sex has been specifically discussed in a few papers [9, 10, 21, 26, 36, 39]. Park et al. called sex the strongest factor that influences the diameter of the gracilis tendon (r = − 0.319), with men having a taller diameter than women [26]. Pinheiro et al. [28] studied eighty patients receiving a quadruple ACL reconstruction. Sex, height, leg length, thigh length, weight, thigh diameter, and the diameter of the harvested graft showed significant correlations. Women presented smaller grafts than men. Men above a height of 180 cm showed a higher percentage of a graft of a diameter greater than 9 mm than women. Ma et al. came to a similar conclusion with the mean graft diameter of 8.1 ± 0.8 (male) vs. 7.5 ± 0.6 mm (female) (*p* < 0.01), reviewing 536 patients with an ACL reconstruction conducted with hamstring grafts [22]. Furthermore, Thomas et al. suggested to include the factor sex into the preoperative planning [32]. In this current study, significant differences between males and females have been examined concerning the CSA of the ST and GT, the length of the ACL, and its tibial and femoral insertion sites. Thus, our findings support the distinct sex influence of the above-mentioned publications.

Most papers in the literature, which investigated the correlation between age and size of grafts, included only a small range of the participants’ age, whereas our study involves a wide range of patients’ ages (10–80 years). Leiter et al. considered patients with a mean age of 27.8 (11.4 SD) years [21], Zakko et al. examined patients with a mean age of 25.1 (10.4 SD) years [39], and Park et al. included patients with a mean age of 29.8 (10.7 SD) years [26], respectively. We showed in this study that age in an adult study group did not correlate with ligament dimensions.

The results of this study show that the anatomy of the knee joint is influenced by many factors. The decision which ACL reconstruction technique is used does not only involve concomitant injuries, but also the assessment of the graft dimensions and the native anatomy. The authors propose a switch from hamstrings to patella autografts or vice versa or allografts for revision of failed ACL reconstruction and the use of allografts to limit donor-site morbidity or using at least one allograft and one autograft for combined ACL and medial collateral ligament (MCL) reconstruction. For some authors, the size of the insertion sites of the ACL is a crucial factor for the decision between a single- or double-bundle technique. A single-bundle technique is chosen over a double-bundle technique, when the total tibial insertion site is ≤ 14 mm [17, 19]. With knowledge of anthropometric data, including measuring of patients MRI, graft dimensions can be preoperatively predicted and individualized ACL reconstruction accordingly planned.

### Limitations

A limiting factor in this study is the fact that anthropometric data were taken from the health record and have not been re-examined at study visit. Certainly not just anthropometric data influence the size and quality of tendons but also physical activity and genetics. We therefore recommend that future studies should include these factors. Furthermore, patients applied to the hospital because of unspecific knee pain. Even though severe damages have been excluded, minor lesions could change the nature of the knee joint. Because of ethical and economical reasons, we refrained from performing MRIs of totally healthy knees. Finally, different dimensions of measurements (graft diameter and CSA) were applied for different graft types.

## Conclusion

Anthropometric data such as weight, height, and sex correlate with dimensions of the quadriceps, patella-, semitendinosus, and gracilis tendons for ACL reconstruction. Patients at risk for small graft dimensions and failure are younger than 20 years and physically active. In these cases, detailed MRI measurements are recommended. Furthermore, measuring the MRI prior to surgery can avoid major complications and should be part of every preoperative planning in the purpose of an individualized ACL reconstruction technique. Anthropometric data can be confidently used in operative planning and decisions for the optimal graft choice.
