# Correction to: Prediction of individual graft for anterior cruciate ligament reconstruction using anthropometric data

**DOI:** 10.1007/s00402-022-04713-w

**Published:** 2022-12-14

**Authors:** Patrick Sadoghi, Veronika Röggla, Hannes Beiglböck, Benjamin Schett, Martin Reschl, Stefan Fischerauer, Patrick Reinbacher, Harald K. Widhalm

**Affiliations:** 1 Department of Orthopaedics and Trauma, Medical University of Graz, Auenbruggerplatz 5, 8036 Graz, Austria; 2 Clinical Division of Traumatology, Department of Orthopaedics and Trauma Surgery, Medical University of Vienna, Währinger Gürtel 18-20, 1090 Vienna, Austria

**Correction to: Archives of Orthopaedic and Trauma Surgery** 10.1007/s00402-022-04682-0

The original version of this article unfortunately contained a mistake. The affiliation details and e-mail address of the following author Veronika Röggla, Hannes Beiglböck, Benjamin Schett, Martin Reschl and Harald K. Widhalm were incorrect.

The correct affiliation and e-mail address should be:

Clinical Division of Traumatology, Department of Orthopaedics and Trauma Surgery, Medical University of Vienna, Währinger Gürtel 18-20, 1090 Vienna, Austria

Veronika Röggla

veronika.roeggla@meduniwien.ac.at

Hannes Beiglböck

hannes.beiglboeck@meduniwien.ac.at

Benjamin Schett

benjamin.schett@meduniwien.ac.at

Martin Reschl

martin.reschl@meduniwien.ac.at

Harald K. Widhalm

harald.widhalm@meduniwien.ac.at

There are typing mistakes in the first column of Tables 2 and 3. The corrected Tables 2 and 3 are given in the following page:

**Table 2:** Descriptive statistics of the measurements of the tendons and the anterior cruciate ligament

	Mean (mm)	SD (mm)	Minimum (mm)	Maximum (mm)
QT-Thickness	4.8	1.1	2.0	9.2
PT-Length	48.3	6.8	30.0	65.4
PT-Thickness	3.2	0.7	1.7	5.7
ST-Diameter	4.8	0.8	2.9	7.1
ST-CSA	13.4	3.6	6.2	27.0
GT-Diameter	4.0	0.8	2.7	8.2
GT-CSA	7.8	2.0	4.0	14.1
ACL-Length	29.8	3.5	22.9	41.4
Tibial Insertion Site	15.8	2.5	10.4	27.8
Femoral Insertion Site	15.2	3.1	9.3	23.0

**Table 3:** Correlation analysis of anthropometric data (height, weight, BMI, sex, age) with autografts for anterior cruciate ligament reconstruction (quadriceps, patella, semitendinosus, and gracilis tendons)

	Height	Weight	BMI	Sex	Age
QT-Thickness	NS	NS	NS	NS	NS
PT-Thickness	NS	*r* = 0.37 (*p* < 0.001)	NS	NS	NS
ST-CSA	*r* = 0.37 (*p* < 0.001)	*r* = 0.43 (*p* < 0.001)	NS	*r* = − 0.44 (*p* < 0.001)	NS
GT-CSA	*r* = 0.35 (*p* < 0.001)	NS	NS	*r* = − 0.34 (*p* < 0.001)	NS
ACL-Length	*r* = 0.62 (*p* < 0.001)	*r* = 0.51 (*p* < 0.001)	NS	*r* = − 0.49 (*p* < 0.001)	NS
Tibial Insertion Site	*r* = 0.42 (*p* < 0.001)	*r* = 0.36 (*p* < 0.001)	NS	*r* = − 0.38 (*p* < 0.001)	NS
Femoral Insertion Site	*r* = 0.54 (*p* < 0.001)	*r* = 0.49 (*p* < 0.001)	NS	*r* = − 0.40 (p < 0.001)	NS

